# Effects of pH alteration on respiratory syncytial virus in human airway epithelial cells

**DOI:** 10.1183/23120541.00404-2022

**Published:** 2023-07-03

**Authors:** Jessica L. Saunders, Ivana A. Daniels, Taiya L. Edwards, Ryan F. Relich, Yi Zhao, Laura A. Smith, Benjamin M. Gaston, Michael D. Davis

**Affiliations:** 1 Division of Pulmonology, Allergy and Sleep Medicine, Riley Hospital for Children, Indianapolis, IN, USA; 2 Herman B. Wells Center for Pediatric Research, Indiana University School of Medicine, Indianapolis, IN, USA; 3 Department of Pathology and Laboratory Medicine, Indiana University School of Medicine, Indianapolis, IN, USA; 4 Department of Biostatistics, Indiana University School of Medicine, Indianapolis, IN, USA

## Abstract

**Background:**

Respiratory syncytial virus (RSV) is a leading cause of respiratory distress and hospitalisation in the paediatric population. Low airway surface pH impairs antimicrobial host defence and worsens airway inflammation. Inhaled Optate safely raises airway surface pH in humans and raises intracellular pH in primary human airway epithelial cells (HAECs) *in vitro*. We aimed to determine whether raising intracellular pH with Optate would decrease infection and replication of RSV in primary HAECs.

**Methods:**

We cultured HAECs from healthy subjects in both air–liquid interface and submerged conditions. We infected HAECs with green fluorescent protein-labelled RSV (GFP-RSV; multiplicity of infection=1) and treated them with Optate or PBS control. We collected supernatant after a 4-h incubation and then every 24 h. We used fluorescence intensity, fluorescent particle counts, plaque assays, Western blots and ELISA to quantitate infection.

**Results:**

In submerged culture, fluorescence intensity decreased in Optate-treated cells (48 h p=0.0174, 72 h p≤0.001). Similarly, Optate treatment resulted in decreased fluorescent particle count (48 h p=0.0178, 72 h p=0.0019) and plaque-forming units (48 h p=0.0011, 72 h p=0.0148) from cell culture supernatant. In differentiated HAECs cultured at ALI, Optate treatment decreased fluorescence intensity (p≤0.01), GFP *via* Western blot and ELISA (p<0.0001), and RSV-fusion protein *via* ELISA (p=0.001). Additionally, RSV infection decreased as Optate concentration increased in a dose-dependent manner (p<0.001).

**Conclusions:**

Optate inhibits RSV infection in primary HAECs in a dose-dependent manner. These findings suggest that Optate may have potential as an inhaled therapeutic for patients with RSV.

## Abstract 2

**Low airway surface pH impairs antimicrobial host defence and worsens airway inflammation. A novel, inhaled, alkaline buffer medication, Optate, raises airway pH and inhibits RSV infection in primary human airway epithelial cells in a dose-dependent manner.** https://bit.ly/4138MAM

## Introduction

Respiratory syncytial virus (RSV) is a member of the Pneumoviridae family that causes airway damage and is the leading cause of severe lower respiratory tract infections in children [1, 2]. In the United States, RSV accounts for >2 million outpatient visits and nearly 60 000 hospitalisations in children aged <5 years annually [3, 4]. Currently, no effective therapies or vaccines exist for RSV, and while preventive antibodies are on the market, they remain restricted to a small group of high-risk infants [5]. A safe treatment for RSV would be beneficial throughout the world.

Airway extracellular pH is acidic during viral respiratory infections and acidic extracellular pH in the airway leads to impaired mucociliary clearance, increased inflammation and decreased host defence [6]. Many viruses, whether enveloped or not, use endocytic entry mechanisms to enter host cells, and an acidic pH serves as a trigger for penetration [7, 8]. Additionally, many viruses require acidic endosomal pH for viral surface protein activation [9]. While RSV was thought to enter the cell in a pH-independent manner *via* direct membrane fusion and release of RSV nucleocapsids into the cytoplasm, recent studies have shown that clathrin function, macropinocytosis and endocytosis play a key role in the virulence of RSV [10, 11]. Specifically, fusion protein cleavage *via* an endosomal protease that may require low pH for activation makes the virus infectious [11–13].

Optate (IND #139144) is a safe, glycine-based, inhaled buffer that alkalinises airway extracellular, intracellular and endosomal pH. We have demonstrated that exposing human airway epithelial cells (HAECs) to Optate also alters endosomal trafficking and inhibits severe acute respiratory syndrome coronavirus 2 (SARS-CoV-2) infection in primary HAECs [14, 15]. Given the dependence of RSV on proteolytic cleavage of the fusion protein in the acidic endosome, we hypothesise that by increasing intracellular pH with Optate we will similarly inhibit RSV infection and replication.

## Material and methods

### Study design

The main objective of this study was to determine whether raising intracellular pH with Optate would decrease infection and replication of RSV in primary HAECs.

### Cell culture and infection model

A compounding pharmacy prepared and assayed Optate (120 mM) for purity, potency, osmolality (target ∼330 mOsmol), pH (target 9.8) and sterility prior to all experiments (IND #139144; Arena District Pharmacy, Columbus, OH, USA).

Primary HAECs from three healthy, nonsmoking donors were grown as described previously under submerged conditions and at air–liquid interface (ALI) at passages 3 or 4 [16–18]. After optimising techniques with one donor under submerged conditions and at ALI, two additional donors were used for all other ALI experiments. Graphs with data from multiple donors are colour coded to allow for easy identification of each donor. Biological replicates are included in the datasets. HAECs were infected with RSV with green fluorescent protein (RSV-GFP; ViraTree, Research Triangle Park, NC, USA; product #R121, RSV-A2) at a multiplicity of infection of 1. Optate (at concentrations used in humans *in vivo* [14, 15]) or control (PBS, pH 7.2) were co-administered with RSV-GFP to HAECs. Negative control groups were cells that were not infected with RSV and were not treated with Optate or PBS. Supernatant was harvested and stored at −80°C after a 4-h incubation period and then every 24 h for 3 days for submerged cells and up to 10 days as needed to achieve optimal infection for cells cultured at ALI. Submerged cells were treated with Optate or PBS daily with media change, while ALI cells were treated apically for 20 min twice daily.

### Quantifying viral infection by fluorescence intensity

Fluorescence intensity quantification was calculated using ImageJ (National Institutes of Health, Bethesda, MD, USA; https://imagej.nih.gov/ij/) on microscopic images obtained with the EVOS M5000 microscope (Thermo Fisher Scientific, Carlsbad, CA, USA) as described previously [14]. Images were taken while microscopy was focused on the most apical layer of epithelial cells every 24 h for either 3 days (submerged cells) or up to 10 days (cells cultured at ALI) using the Invitrogen GFP Light Cube (Thermo Fisher Scientific) prior to daily treatment with PBS or Optate. Fluorescence images were uploaded to ImageJ, image fields were selected, images were processed by subtracting the background using default settings, fluorescence intensity was analysed and average fluorescence intensity was compared between all groups. This technique reflects the level of RSV-GFP infection by quantifying fluorescence from the GFP in infected cells [19].

### Fluorescent particle counting and plaque assays

Vero E6 (African green monkey kidney cell line; ATCC, Manassas, VA, USA) were plated on the day prior to the experiment. Plaque assays were performed as described previously using the stored supernatant from the previous experiments outlined earlier [14]. Fluorescent particles were counted using fluorescence microscopy with Invitrogen GFP Light Cube on day 5 and results are reported as focus-forming units per millilitre.

### Detection of GFP and RSV fusion glycoprotein by ELISA

After 5 days of infection with RSV and treatment with either Optate or PBS, growth medium was removed and cells were rinsed in PBS. Cells were then lysed by adding 50 μL of 1X Cell Extraction Buffer PTR (GFP *in vitro* CatchPoint SimpleStep ELISA) and phosphatase inhibitor, which was directly applied to ALI filters. Cells were scraped off the filters using a flat pipette tip and lysate was transferred to a microfuge tube. Samples were centrifuged at 18 000×*g* for 20 min at 4°C. The supernatants were transferred into clean tubes and the pellets were discarded. Samples were stored at −80°C until the assay was performed. The sample protein concentration in the extract was quantified using a protein assay (Pierce BCA Protein Assay; Thermo Fisher Scientific). Samples were diluted to desired concentration in 1X Cell Extraction Buffer PTR immediately prior to use. GFP *in vitro* CatchPoint SimpleStep ELISA (Abcam, Cambridge, UK) was used to quantify GFP and RSV was quantified using RSV (A2) Fusion Glycoprotein (RSV-F) ELISA Kit (SinoBiological, Wayne, PA, USA). Technical replicates were averaged. Results were plotted on a four-parameter logistic regression model per package instructions, and concentrations were calculated [20].

### Immunoblots

Using the same protocol as that for ELISA, cells were lysed in 1X Cell Extraction Buffer PTR. Capillary electrophoresis was performed on the automated Jess system as described previously (ProteinSimple, San Jose, CA, USA) [14]. Briefly, 0.25 μg·μL^−1^ lysate was plated and run according to the manufacturer's recommendations. Antibodies to GFP (part number 2956T; Cell Signaling Technology, Boston, MA, USA) were used. Compass software (ProteinSimple) generated digitally rendered bands based on chemiluminescence electrophoretogram to quantify GFP.

### Intracellular pH assays

Intracellular pH was evaluated as described previously using two fluorescent dyes: 2′,7′-bis-(2-carboxyethyl)-5-(and-6)-carboxyfluorescein, acetoxymethyl ester (BCECF-AM; Invitrogen, Carlsbad, CA, USA) and pHrodo Red (Invitrogen) [14]. After washing with PBS, cells were treated with Optate (clinical solution, 1:1 dilution in medium) or PBS.

### Analysis

All statistical analyses were calculated using GraphPad Prism (GraphPad Software, San Diego, CA, USA) and R (R Core Team, Vienna, Austria). To evaluate differences in fluorescence intensity, fluorescent particle counts, viral plaques and RSV-F and GFP protein quantities between negative control and treatment groups, a two-sample two-tailed t-test was used for Gaussian-distributed data and Wilcoxon rank-sum test was used for non-Gaussian-distributed data (supplementary table S1). For cases with more than two groups, robust ANOVA models were considered with pairwise Tukey's test. With multiple time points, the tests were performed for each time point separately. To study the association with pH, robust linear regression models were fitted. For ELISA data, a four-parameter logistic regression model was used [20]. A p-value <0.05 was considered statistically significant.

### Biosafety and ethics statement

All experiments were conducted at Indiana University (Indianapolis, IN, USA) in a biosafety level 2 laboratory (IBC# IN-1127). Primary cells were obtained under Indiana University institutional review board protocol #1408855616. All subjects provided informed consent.

## Results

### Optate treatment inhibits RSV infection in submerged cells

**FIGURE 1:**
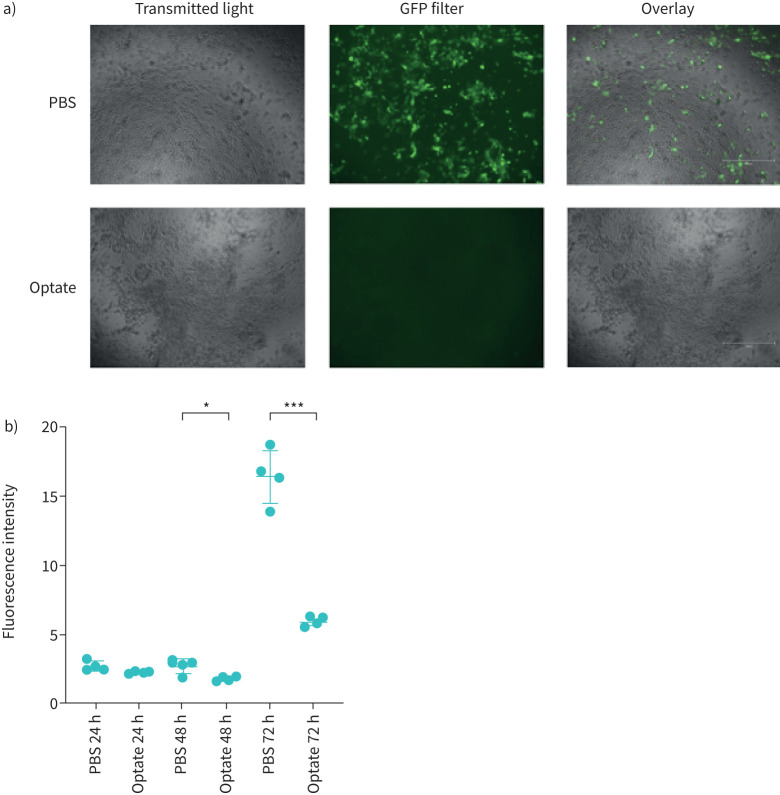
Respiratory syncytial virus infection is significantly decreased in submerged human airway epithelial cells treated with Optate compared to PBS control. a) Fluorescence microscopy images taken using transillumination and green fluorescent protein (GFP)-filter at 72 h to demonstrate the difference in infection between Optate-treated cells compared to PBS control. No difference was noted in cell viability between the two groups. b) ImageJ software was used to quantify fluorescence intensity, which is significantly decreased in the Optate-treated groups at the 48-h (n=4, p=0.0174) and 72-h (n=4, p<0.001) time points compared to control. *: p≤0.05, ***: p≤0.001.

**FIGURE 2:**
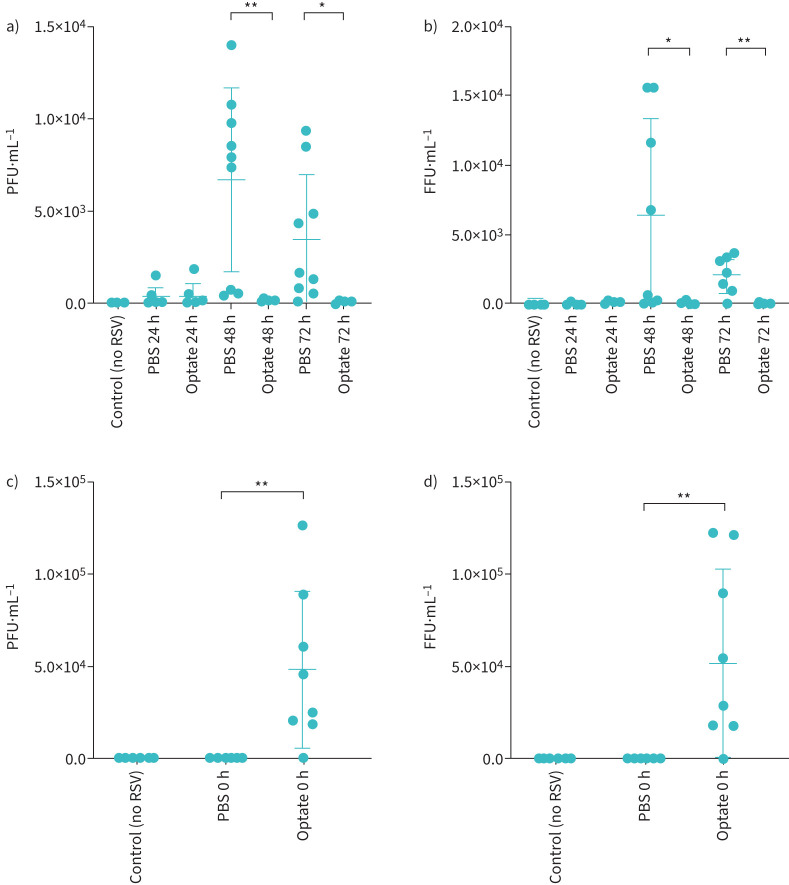
Respiratory syncytial virus (RSV) is significantly decreased in the supernatant of submerged human airway epithelial cells treated with Optate compared to control. a) Plaque-forming units (PFUs) are significantly reduced in Optate-treated cell supernatant at 48-h (n=9, p=0.0011) and 72-h (n=9, p=0.0148) time points compared to control. b) Fluorescent particle count by fluorescence microscopy performed on day 5 of plaque assays prior to fixing the plates was significantly reduced in cell supernatant from Optate-treated cells at the 48-h (n=7, p=0.0178) and 72-h (n=7, p=0.0019) time points compared to control. Fluorescent particles were counted and are reported as focus-forming units per mililitre (FFU·mL^−1^). c) PFUs are significantly increased (n=8, p=0.0011) in the supernatant of Optate-treated cells immediately following the incubation period. d) FFUs are significantly increased (n=7, p=0.0017) in the supernatant of Optate-treated cells immediately following the incubation period. *: p≤0.05, **: p≤0.01.

In submerged HAECs, RSV infection was lower in cells treated with Optate compared to control at 48 h and 72 h. Figure 1a shows fluorescence microscopy images taken 72 h post-infection and demonstrates the difference in RSV infection between Optate-treated cells and PBS control. Figure 1b demonstrates a significant decrease in fluorescence intensity quantified using ImageJ during primary infection (48 h n=4, p=0.0174; 72 h n=4, p<0.0001). These results were confirmed by fluorescent particle count and plaque assays from supernatant (48 h n=9, p=0.0178; 72 h n=9, p=0.0019 and 48 h n=7, p=0.0011; 72 h n=7, p=0.0148, respectively; figure 2a and b). Immediately following the incubation period, supernatant collected from Optate-treated cells had a significantly higher viral load indicated by increased fluorescent particle counts (n=7, p=0.0017) and particle-forming units (PFUs) (n=8, p=0.0011) compared to PBS control (figure 2c and d).

### Inhibition of RSV by optate is dose-dependent

**FIGURE 3:**
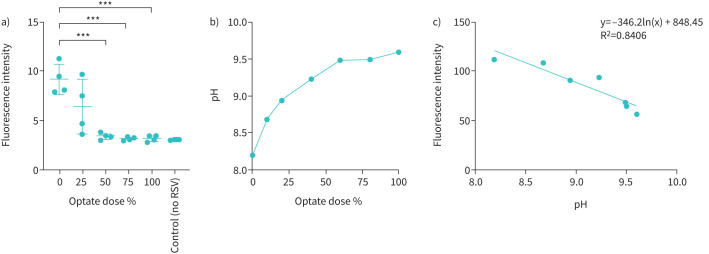
Optate inhibits respiratory syncytial virus (RSV) infection and alters intracellular pH in a dose-dependent manner. a) Fluorescence intensity is decreased in a dose-dependent manner at 72 h post-infection with green fluorescent protein-RSV. Fluorescence intensity was quantified using ImageJ 72 h post-infection. Optate significantly decreased RSV infection to the level of the negative control group at doses ≥50% (n=4, p≤0.001). b) pH decreases as the dose (buffer content) of Optate decreases. Per a), Optate is most effective at doses >50%, which are above pH 9.2. Dose is defined as percentage of glycine buffer in Optate, with 100% representing 120 mM, 75% representing 90 mM, 50% representing 60 mM, and 25% representing 30 mM. c) Optate pH strongly correlates with intracellular fluorescence intensity in cells treated with pH-sensitive intracellular dye pHrodo Red. ***: p≤0.001.

RSV infection in submerged HAECs decreased in a dose-dependent manner, as shown in figure 3. Figure 3a shows the decrease in viral fluorescence intensity quantified using ImageJ. Optate significantly decreased RSV infection to the level of the negative control group at doses ≥50% (n=4, p<0.001). Furthermore, pH decreased as the dose of Optate decreased (figure 3b). Finally, Optate pH strongly correlated with intracellular fluorescence intensity in cells treated with pH-sensitive intracellular dye pHrodo Red (R^2^=0.8406, figure 3c).

### Optate treatment inhibits RSV infection in organotypic, differentiated primary HAECs

**FIGURE 4:**
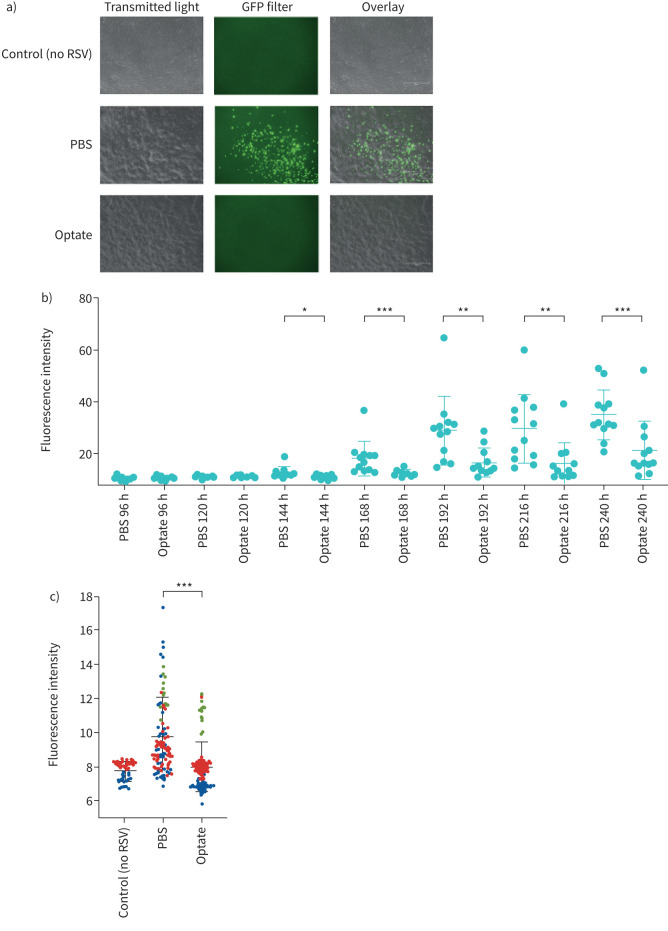
Optate inhibits respiratory syncytial virus (RSV) infection in human airway epithelial cells (HAECs) cultured at air–liquid interface (ALI). a) Fluorescence microscopy images taken using transillumination and green fluorescent protein (GFP)-filter at 72 h to demonstrate the difference in infection between Optate-treated cells compared to PBS control. No difference was noted in cell viability between the two groups. b) HAECs from a single donor were used to determine time-course of infection at ALI. Fluorescence intensity is significantly decreased in the Optate-treated groups between the 144-h and 240-h time points compared to control (n=12). c) Fluorescence intensity is significantly decreased in the Optate-treated groups during primary infection of HAECs from three donors. Negative control included for reference of autofluorescence. (n=96). Data are presented as mean±sem. *: p≤0.05, **: p≤0.01, ***: p≤0.001.

**FIGURE 5:**
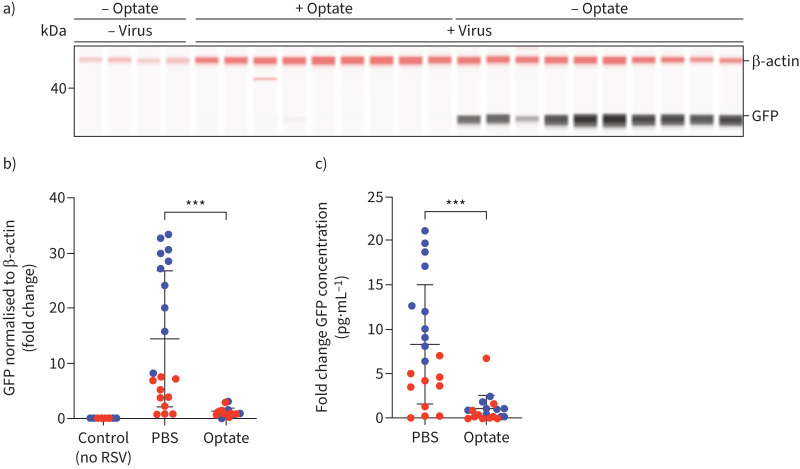
Optate inhibits respiratory syncytial virus (RSV) infection in human airway epithelial cells (HAECs) cultured at air–liquid interface as evidenced by a reduction in green fluorescent protein (GFP) *via* Jess and ELISA. a) Western blot demonstrating a reduction of GFP in samples treated with Optate compared to those treated with PBS. b) GFP is significantly reduced in cells treated with Optate compared to PBS control using Jess (n=20, p<0.0001). HAECs from two different donors were used to verify this observation. Samples were normalised to β-actin for comparison and then datasets were normalised to reflect fold change. c) GFP ELISA shows a significant reduction in GFP production in cells treated with Optate compared to PBS control (n=20, p<0.0001). HAECs from two different donors were used to verify this observation, and data were normalised to reflect fold change instead of reporting protein concentrations, due to significant variation in relative concentrations between the donor cells. ***: p≤0.001.

**FIGURE 6:**
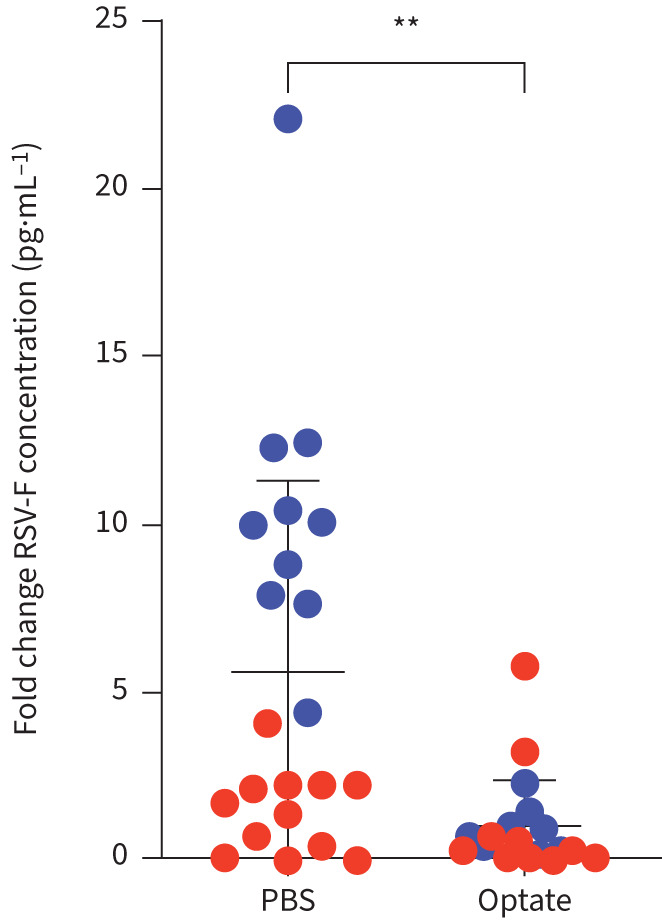
Optate inhibits respiratory syncytial virus (RSV) infection in human airway epithelial cells (HAECs) cultured at air–liquid interface as evidenced by a reduction in RSV-fusion protein (RSV-F) by ELISA (n=22, p=0.001). HAECs from two different donors were used to verify this observation, and data were normalised to reflect fold change instead of reporting protein concentrations due to significant variation in relative concentrations between the donor cells.

In primary HAECs cultured at ALI, fluorescence intensity decreased in cells treated with Optate compared to control during primary infection (p≤0.05; figure 4a, b and c). Cells were treated twice daily with Optate or PBS control and fluorescent images were obtained every 24 h post-infection. In addition, there was a significant decrease in the amount of GFP quantified using Jess (figure 5a and b) and ELISA (figure 5c), along with a significant reduction in RSV-F protein quantified using ELISA (figure 6).

## Discussion

These experiments demonstrate that Optate inhibits RSV infection and replication in primary HAECs grown under both submerged conditions and at ALI. Furthermore, RSV infection decreased with increasingly concentrated Optate administration. Our group has shown previously that treatment with Optate raises both intracellular and extracellular pH, is not cytotoxic, is safe and well tolerated in humans, and prevents SARS-CoV-2 replication in primary HAECs (supplementary video S1) [14, 15].

**FIGURE 7:**
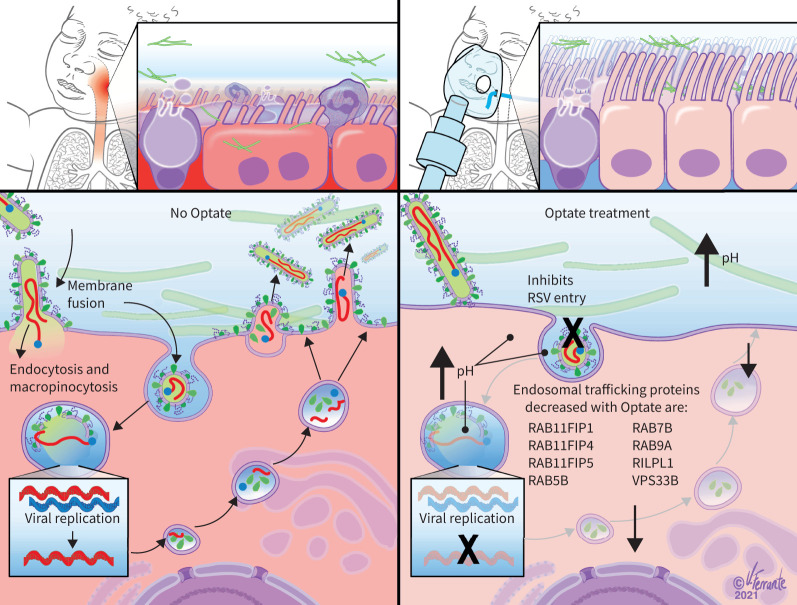
Respiratory syncytial virus (RSV) inhibition by Optate. RSV is an enveloped virus that can enter the host cell *via* direct membrane fusion, endocytosis and macropinocytosis. Although it has several mechanisms to enter the host cell, replication relies on the endosome for cleavage of the fusion protein and thus its infectivity. Optate raises the extracellular, intracellular and endosomal pH and alters endosomal trafficking. We speculate that Optate decreases RSV entry into the host cell *via* altered endosomal trafficking.

Of note, fluorescent particle count and PFUs in the plaque assays of supernatant from Optate-treated cells increased immediately after RSV inoculation (figure 2c and d), despite the fact that Optate reduced intracellular RSV infection and replication at later time points (figure 2a and b). This suggests decreased uptake of the virus into the cells. Altered endosomal trafficking caused by Optate may explain the decrease in viral entry into the cell [14]. At later time points, a decrease in fluorescent particle count and PFUs in plaques assays of Optate-treated cells suggests that Optate also inhibits RSV replication within the cell. Figure 7 illustrates our proposed mechanisms for RSV inhibition by Optate.

Our study has several limitations. All the data presented from our cell culture models *in vitro* may not translate to *in vivo* systems and disease processes. In addition, our use of fluorescence microscopy to quantify infection may be confounded by the presence of multiple layers of cells in ALI. Furthermore, our current results support the hypothesis that Optate inhibits RSV through alterations in endosomal trafficking, but further studies are needed to confirm this mechanism. One of the challenges of studying airway pH is that exact measurements of pH are difficult to obtain and require direct sampling during bronchoscopy. Even then, the act of sampling airway pH may affect the results. Exhaled breath condensate pH and changes in exhaled nitric oxide are therefore often used as surrogate markers of airway pH. Although a normal range of pH has been established a using exhaled breath condensate, exact airway pH measurements are unknown. Using exhaled breath condensate pH and changes in exhaled nitric oxide, we know that Optate transiently raises airway pH in humans *in vivo*, but to what extent is unknown [15].

In conclusion, Optate reduces RSV infection in primary HAECs. Our results suggest that this is due to increased intracellular pH [14]. These findings suggest that Optate might be the focus of additional studies aimed at developing a treatment for patients with RSV. These studies could include further mechanistic experiments in addition to clinical studies.

## Supplementary material

10.1183/23120541.00404-2022.Supp1 **Please note:** supplementary material is not edited by the Editorial Office, and is uploaded as it has been supplied by the author.
TABLE S1 Description of Gaussian distribution and statistical analysis used in each figure 00404-2022.supplement
VIDEO S1 Ciliary motion prior to Optate 00404-2022.supplement
VIDEO S2 Ciliary motion with Optate, 30 min 00404-2022.supplement2
VIDEO S3 Ciliary motion with Optate, 1 h 00404-2022.supplement3
VIDEO S4 Ciliary motion with Optate, 2 h 00404-2022.supplement4
VIDEO S5 Ciliary motion with Optate, 3 h 00404-2022.supplement5
VIDEO S6 Ciliary motion with Optate, 4 h 00404-2022.supplement6
VIDEO S7 Ciliary motion with Optate, 6 h 00404-2022.supplement7
VIDEO S8 Ciliary motion with Optate, 8 h 00404-2022.supplement8
