# Supplementary material for: Effects of pH alteration on respiratory syncytial virus in human airway epithelial cells
Source: ERJ Open Res. 2023 Jul 3;9(4):00404-2022. doi: 10.1183/23120541.00404-2022 (PMC10351676; doi:10.1183/23120541.00404-2022)
Supplement: Supplementary file 1 [file 00404-2022.supplement.pdf]

Table S1: Description of Gaussian distribution and statistical analysis used in each figure.

Figure	Gaussian Distribution	Parametric vs Nonparametric Test
1B	Yes	Unpaired t test
2A	Yes	Unpaired t test
2B	Yes	Unpaired t test
2C	No	Wilcoxon Rank-Sum Test
2D	No	Wilcoxon Rank-Sum Test
4B	No	Wilcoxon Rank-Sum Test
4C	No	Wilcoxon Rank-Sum Test
5B	Yes	Unpaired t test
5C	No	Wilcoxon Rank-Sum Test
6	No	Wilcoxon Rank-Sum Test

Supplement Figure 1: Ciliary beating is unaffected by exposure to Optate. Ciliary beating was recorded prior to administration of Optate, 30 minutes after administration, and hourly until removal of apically administered Optate at 8 hours. No changes in ciliary beating were noted.
